# Upregulation of IL‐8, osteonectin, and myonectin mRNAs by intermittent hypoxia via OCT1‐ and NRF2‐mediated mechanisms in skeletal muscle cells

**DOI:** 10.1111/jcmm.17618

**Published:** 2022-12-01

**Authors:** Shin Takasawa, Ryogo Shobatake, Asako Itaya‐Hironaka, Mai Makino, Tomoko Uchiyama, Sumiyo Sakuramoto‐Tsuchida, Yoshinori Takeda, Hiroyo Ota, Akiyo Yamauchi

**Affiliations:** ^1^ Department of Biochemistry Nara Medical University Nara Japan; ^2^ Department of Neurology Nara Medical University Nara Japan; ^3^ Department of Neurology Nara City Hospital Nara Japan; ^4^ Department of Diagnostic Pathology Nara Medical University Nara Japan; ^5^ Department of Respiratory Medicine Nara Medical University Nara Japan

**Keywords:** intermittent hypoxia, myokine(s), NRF2, OCT1, sleep apnoea syndrome

## Abstract

Sleep apnoea syndrome is characterized by recurrent episodes of oxygen desaturation and reoxygenation (intermittent hypoxia [IH]) and is a risk factor for insulin resistance/Type 2 diabetes. The induction of insulin resistance in skeletal muscle is a key phenomenon to develop diabetes. However, the mechanisms linking IH stress and insulin resistance remain elusive. We exposed human RD and mouse C2C12 muscle cells to normoxia or IH and measured their mRNA levels by real‐time RT‐PCR. We found that IH significantly increased the mRNA and protein levels of muscle‐derived insulin resistance‐factors (myokines) such as IL‐8, osteonectin (ON), and myonectin (MN) in muscle cells. We further analysed the IH‐induced expression mechanisms of *IL‐8*, *ON*, and *MN* genes in muscle cells. Deletion analyses of the human myokine promoter(s) revealed that the regions −152 to −151 in *IL‐8*, −105 to −99 in *ON*, and − 3741 to −3738 in *MN* promoters were responsible for the activation by IH in RD cells. The promoters contain consensus transcription factor binding sequences for OCT1 in *IL‐8* and *MN* promoters, and for NRF2 in *ON* promoter, respectively. The introduction of siRNA for OCT1 abolished the IH‐induced expression(s) of *IL‐8* and *MN* and siRNA for NRF2 abolished the IH‐induced expression of *ON*.

## 1 INTRODUCTION

Sleep apnoea syndrome (SAS) is a common disorder characterized by repetitive episodes of oxygen desaturation during sleep, by development of daytime sleepiness, and by deterioration of quality of life.^1^, ^2^ SAS is caused by the obstruction of the upper airway, and moderate to severe cases of SAS affect 10%–17% of men and 3%–9% of women aged between 30 and 70 years.^3^ During sleep, the repeated upper airway obstruction in SAS patients can cause serious recurrent apnoea, and it exposes these patients to alternating low oxygen pressure and normal oxygen pressure levels, that is, intermittent hypoxia (IH).^4^ SAS is associated with many systemic complications, such as obesity, type 2 diabetes,^4^, ^5^, ^6^, ^7^ dyslipidaemia,^4^, ^7^ cardiovascular diseases (e.g., hypertension, coronary disease, heart failure, and stroke),^8^ pulmonary hypertension,^5^, ^9^ neurocognitive deficits,^10^, ^11^ depression,^12^ and impaired memory.^13^

Recently, several proteins called myokines, which are exclusively or predominantly secreted in muscle tissue, were established as directly affecting glucose and lipid metabolism.^14^, ^15^, ^16^ For example, some myokines such as interleukin (IL)‐6, IL‐8, IL‐15, and tumour necrosis factor‐α (TNFα) were found to express highly in muscles of Type 2 diabetes patients^17^ decreasing insulin sensitivity.^18^ Moreover, newly diagnosed Type 2 diabetes and impaired glucose tolerance subjects had higher circulating erythroferrone (ERFE)/myonectin (MN) concentrations than normal subjects; also, plasma MN is correlated positively with waist/hip ratio, body fat percentage, triglyceride, fasting blood glucose, 2‐hour blood glucose after glucose overload, fasting insulin, haemoglobin A1c, and with the homeostasis model assessment of insulin resistance.^19^ However, the IH‐induced changes in the levels of these myokines in myocytes remain elusive.

In the present study, using muscle cells and an in vitro IH system, which is a controlled gas delivery system that regulates the flow of nitrogen and oxygen to generate IH, we investigated the direct effect of IH, a hallmark of SAS, on the gene expression levels of *IL‐6*, *IL‐8*, *IL‐15*, *TNFα*, *myostatin* (*MSTN*), *brain‐derived neurotrophic factor* (*BDNF*), *IRISIN*, *Decorin* (*DCN*), *secreted protein acidic and cysteine rich* (*SPARC*)*/osteonectin* (*ON*), *ERFE/MN*, *glucose transporter type 4* (*GLUT4*), *mitogen‐activated protein kinase 14* (*MAPK14*), *phosphatidylinositol 3‐kinase regulatory subunit β* (*PI3KR2*), and *sirtuin 2* (*SIRT2*). A significant increase in the mRNA levels of *IL‐8*, *ON*, and *MN* in two different muscle cells in response to IH treatment was detected. We also showed that the IH‐induced upregulation of *IL‐8* and *MN* requires octamer binding transcription factor 1 (OCT1) and that the IH‐induced upregulation of *ON* requires nuclear factor erythroid 2‐related factor 2 (NRF2) as transcriptional factors.

## 2 MATERIALS AND METHODS

### 2.1 Cell culture

Mouse C2C12 skeletal myoblasts were purchased from Riken BioResource Research Center (Tsukuba, Japan). The cells were maintained in Dulbecco's Modified Eagle Medium (DMEM) (FUJIFILM Wako Pure Chemical Corporation) containing 10% (v/v) fetal calf serum (FCS), 100 units/ml penicillin G (FUJIMILM Wako), and 100 μg/ml streptomycin (FUJIFILM Wako). Once a suitable of cell proliferation was achieved (90% confluency), the medium was changed to the differentiation medium (DMEM containing 2% [v/v] horse serum, 100 units/ml penicillin G, and 100 μg/ml streptomycin). Human rhabdomyosarcoma RD cells were purchased from the Japanese Collection of Research Bioresources Cell Bank (JCRB). The cells were grown in Eagle's Minimum Essential Medium (E‐MEM) medium (FUJIFILM Wako) containing 10% (v/v) FCS, 100 units/ml penicillin G, and 100 μg/ml streptomycin. The cells were exposed to either normoxia (21% O_2_, 5% CO_2_, and balanced N_2_) or intermittent hypoxia (IH: 64 cycles of 5 min of sustained hypoxia [SH: 1% O_2_, 5% CO_2_, and balanced N_2_] and 10 min of normoxia) using a custom‐designed, computer‐controlled incubation chamber attached to an external O_2_‐CO_2_‐N_2_ computer‐driven controller (O_2_ programmable control, 9200EX, Wakenbtech CO., Ltd), as described.^4^, ^20^, ^21^, ^22^, ^23^, ^24^ These conditions are similar to those reported in studies involving patients with severe SAS, wherein patients are repeatedly exposed to severe hypoxemia followed by mild hypoxemia or normoxia (i.e., IH). We previously reported that the magnitude of IH expressed by SpO_2_ fluctuated between 75%–98% and 50%–80% in SAS,^4^ which was nearly equivalent to the medium condition in the present study.

### 2.2 Real‐time reverse transcriptase‐polymerase chain reaction (RT‐PCR)

Total RNA was isolated from C2C12 and RD cells using an RNeasy Protect Cell Mini Kit (Qiagen, Hilden, Germany), and cDNA was synthesized from the total RNA as template by using a High Capacity cDNA Reverse Transcription Kit (Applied Biosystems) as described.^4^, ^20^, ^21^, ^22^, ^23^, ^24^, ^25^ Real‐time PCR was performed using an SYBR® Fast qPCR kit (KAPA Biosystems) and a Thermal Cycler Dice Real Time System (Takara Bio). All PCR primers were synthesized by Nihon Gene Research Laboratories, Inc. (NGRL); the primer sequences for each primer set are described in Table 1. PCR was performed as follows: an initial step at 95°C for 3 min followed by 40 cycles of 95°C for 3 sec and 60°C for 20 sec for *β‐actin*, *rat insulinoma gene (Rig)/RpS15*, *IL‐6*, *CCL2*, *IL‐8*, *IL‐15*, *TNFα*, *MSTN*, *BDNF*, *IRISIN*, *DCN*, *SPARC/ON*, *ERFE/MN*, *NOX2*, *GLUT4*, *MAPK14*, *PI3KR2*, and *SIRT2*. The mRNA expression levels were normalized to the mRNA level of *Rig/RpS15* in mouse samples or of *β‐actin* in human samples.

**TABLE 1:** PCR primers for real‐time RT‐PCR.

Target mRNA	Primer sequence (Accession number: Position)
Human	
*GLUT4*	5′‐CCCCCTCAGCAGCGAGTGA‐3′ (NM_001042.3: 260–278)
	5′‐GCACCGCCAGGACATTGTTG‐3′ (NM_001042.3: 559–578)
*MAPK14*	5′‐CGAGCGTTACCAGAACCTGT‐3′ (NM_001315.3: 413–432)
	5′‐GGAGAGCTTCTTCACTGCCA‐3′ (NM_001315.3: 499–518)
*PI3KR2*	5′‐ATGGCACCTTCCTAGTCCGAGA‐3′ (NM_005027.4: 1601–1622)
	5′‐CTCTGAGAAGCCATAGTGCCCA‐3′ (NM_005027.4: 1707–1728)
*SIRT2*	5′‐CAGACCCCTCTCACCCTCTG‐3′ (NM_012237.4: 108–127)
	5′‐GTCATAGAGGCCGGTGGATG‐3′ (NM_012237.4: 393–412)
*IL‐6*	5′‐GGTACATCCTCGACGGCATC‐3′ (NM_000600.5: 236–255)
	5′‐GCCTCTTTGCTGCTTTCACAC‐3′ (NM_000600.5: 294–314)
*IL‐8*	5′‐TAGCAAAATTGAGGCCAAGG‐3′ (NM_000584.4: 683–702)
	5′‐GGACTTGTGGATCCTGGCTA‐3′ (NM_000584.4: 868–887)
*IL‐15*	5′‐ATGGATGGCTGCTGGAAAC‐3′ (NM_000585.5: 313–331)
	5′‐TGCACTGAAACAGCCCAAAA‐3′ (NM_000585.5: 491–510)
*TNFα*	5′‐CTTCTCCTTCCTGATCGTGG‐3′ (NM_000594.4: 282–301)
	5′‐TCTCAGCTCCACGCCATT‐3′ (NM_000594.4: 529–537)
*MSTN*	5′‐TGGTCATGATCTTGCTGTAACCTT‐3′ (NM_005259.3: 832–855)
	5′‐TGTCTGTTACCTTGACCTCTAAAAACG‐3′ (NM_005259.3: 885–911)
*BDNF*	5′‐CAGGGGCATAGACAAAAG‐3′ (NM_170735.6: 1682–1699)
	5′‐CTTCCCCTTTTAATGGTC‐3′ (NM_170735.6: 1817–1834)
*IRISIN*	5′‐AGGTGCTGATCATCGTCGT‐3′ (NM_001171941.3: 454–472)
	5′‐CCTCTGCAGTCCAGGGATT‐3′ (NM_001171941.3: 679–697)
*DCN*	5′‐CGCCTCATCTGAGGGAGCTT‐3′ (NM_001920.5: 999–1018)
	5′‐TACTGGACCGGGTTGCTGAA‐3′ (NM_001920.5: 1184–1203)
*SPARC/ON*	5′‐CACGGCAAGGTGTGCGAG‐3′ (NM_003118.4: 299–316)
	5′‐AGAAGTGGCAGGAAGAGTCGAA‐3′ (NM_003118.4: 419–440)
*ERFE/MN*	5′‐AGTCCCGGTGCCAGCGCAA‐3′ (NM_001291832.2: 904–922)
	5′‐CGCCCAGGAGGACAGCACTGAA‐3′ (NM_001291832.2: 1077–1098)
*β‐Actin*	5′‐GCGAGAAGATGACCCAGA‐3′ (NM_001101.5: 431–448)
	5′‐CAGAGGCGTACAGGGATA‐3′ (NM_001101.5: 503–520)
Mouse	
*Il‐6*	5′‐ACAACCACGGCCTTCCCTACTT‐3′ (NM_031168.2: 139–160)
	5′‐CAGGATTTCCCAGCGAACATGTG‐3′ (NM_031168.2: 245–264)
*Il‐8*	5′‐CAGAAAGGAAGTGATAGCAGTCCCA‐3′ (NM_011339.2: 211–235)
	5′‐CAAAGTGTCTAGAGGTCTCCCGAA‐3′ (NM_011339.2: 441–464)
*Il‐15*	5′‐ACATCCATCTCGTGCTACTTGT‐3′ (NM_008357.2: 537–558)
	5′‐GCCTCTGTTTTAGGGAGACCT‐3′ (NM_008357.2: 629–649)
*Tnfα*	5′‐CCTCCCTCTCATCAGTTCTA‐3′ (NM_013693.3: 368–387)
	5′‐ACTTGGTGGTTTGCTACGAC‐3′ (NM_013693.3: 450–469)
*Mstn*	5′‐ACTGGAATCCGATCTCTGAAACTT‐3′ (NM_010834.3: 688–711)
	5′‐GACCTCTTGGGTGTGTCTGTCAC‐3′ (NM_010834.3: 898–920)
*Bdnf*	5′‐ATTAGCGAGTGGGTCACAGC‐3′ (NM_007540.4: 1097–1116)
	5′‐TCAGTTGGCCTTTGGATACC‐3′ (NM_007540.4: 1180–1199)
*Irisin*	5′‐TGAAGTGGTCATTGGCTTTGC‐3′ (NM_027402.4: 248–268)
	5′‐GCGGGTGGTGGTGTTCAC‐3′ (NM_027402.4: 318–335)
*Dcn*	5′‐GCTGCGGAAATCCGACTTC‐3′ (NM_001190451.2: 791–809)
	5′‐TTGCCGCCCAGTTCTATGAC‐3′ (NM_001190451.2: 831–850)
*Sparc/On*	5′‐AAACATGGCAAGGTGTGTGA‐3′ (NM_009242.5: 535–554)
	5′‐AAGTGGCAGGAAGAGTCGAA‐3′ (NM_009242.5: 658–677)
*Erfe/Mn*	5′‐GGTGGATCGGCGTGCGTTG‐3′ (NM_173395.2: 654–672)
	5′‐TCCCGGGGTCGTGTTGGTC‐3′ (NM_173395.2: 830–848)
*Rig/RpS15*	5′‐ACGGCAAGACCTTCAACCAG‐3′ (NM_009091.2: 343–362)
	5′‐ATGGAGAACTCGCCCAGGTAG‐3′ (NM_009091.2: 392–412)

### 2.3 Measurement of IL‐8, ON, and MN in culture medium by ELISA

RD cells (1 × 10^5^ cells/ml in E‐MEM containing 10% (v/v) FCS, 100 units/ml penicillin G, and 100 μg/ml streptomycin) were seeded in 24‐well plate. Cells were exposed to either normoxia or IH for 24 h; the culture medium was collected, and the concentrations of IL‐8, SPARC (ON), and ERFE (MN) were measured by using an IL‐8 ELISA Kit (Proteintech Group Inc), a Human/Pig Osteonectin EIA Kit (Takara Bio), and an Erythroferrone (ERFE) ELISA kit (CLOUD‐CLONE Corp), respectively.

### 2.4 RNA interference

Small interfering RNA (siRNA) directed against human *OCT1* and *NRF2* were synthesized by NGRL. The sense sequences of siRNA for human *OCT1* and *NRF2* were 5′‐CCUCGGAAGAGAUCACUAUtt‐3′ (corresponding to the 1295–1313 of NM_002697.4) and 5′‐CCCAUUGAUGUUUCUGAUCUAtt‐3′ (corresponding to the 1097–1117 of NM_006164.5), respectively. The Silencer® Select Human Scrambled siRNA was purchased from Ambion® and was used as a control. Transfection of siRNAs into RD cells was carried out using Lipofectamine® RNAiMAX Transfection Reagent (Life Technologies). Cells were transfected with 5 pmol each of siRNA in a 24‐well culture dish as described.^20^, ^21^, ^22^, ^23^, ^24^, ^25^

### 2.5 Construction of reporter plasmid and luciferase assay

Reporter plasmids were prepared by inserting the promoter fragments of human *IL‐8* (−1914 to +98, −521 to +98, −194 to +98, −178 to +98, −152 to +98, −151 to +98, −149 to +98, −147 to +98, −141 to +98, −133 to +98, −115 to +98, and − 95 to +98), *ON* (−2702 to +145, −997 to +145, −712 to +145, −516 to +145, −105 to +145, −99 to +145, −92 to +145, −72 to +145, and − 41 to +145), and *MN* (−3741 to −221, −3738 to −221, −3729 to −221, −3721 to −221, −3702 to −221, −3692 to −221, −3634 to −221, −3582 to −221, −3492 to −221, −3249 to −221, and − 3013 to −221) upstream of a firefly luciferase reporter gene in the pGL4.17[*luc2*/Neo] vector (Promega). The reporter plasmids were transfected into human RD cells using Lipofectamine® 3000 (Invitrogen), as described,^20^, ^21^, ^22^, ^23^, ^24^, ^25^ and the cells were exposed to either 64 cycles/24 h of IH, mimicking the myocytes of SAS patients, or to normoxia for 24 h. The cells were harvested, and cell extracts were prepared in Extraction Buffer (0.1 M potassium phosphate, pH 7.8/0.2% Triton X‐100; Life Technologies). To monitor transfection efficiency, we co‐transfected pCMV‐SPORT‐βgal plasmid (Life Technologies) in all experiments at a 1:10 dilution. Luciferase activity was measured using a PicaGene Luciferase Assay System (Toyo‐ink) and was normalized by the β‐galactosidase activity as described previously.^20^, ^21^, ^22^, ^23^, ^24^, ^25^

### 2.6 Data analysis

Results are expressed as mean ± SE. Statistical significance was determined by Student's *t*‐test using the GraphPad Prism software (GraphPad Software).

## 3 RESULTS

### 3.1 Gene expression levels of IL‐8, ON, and MN in muscle cells were increased by IH

**FIGURE 1:**
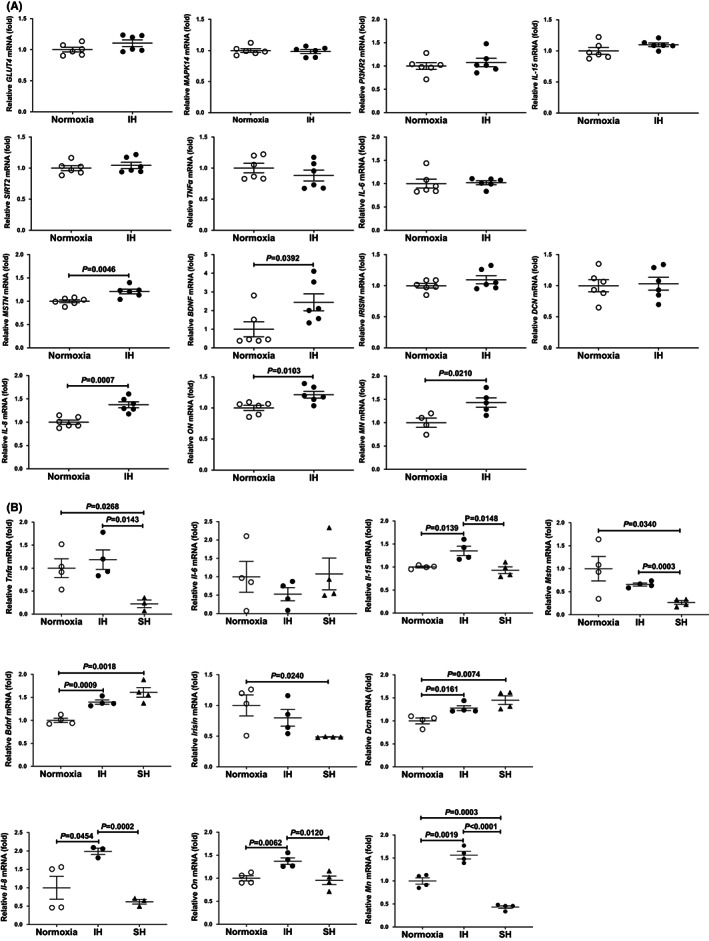
The mRNA levels of *IL‐8*, *ON*, *MN*, *GLUT4*, *MAPK14*, *PI3KR2*, *SIRT2*, *TNFα*, *IL‐6*, *IL‐15*, *MSTN*, *BDNF*, *IRISIN*, and *DCN*. (A) Human RD and (B) differentiated mouse C2C12 cells treated with normoxia, IH, or sustained hypoxia (SH). The mRNA levels were measured by real‐time RT‐PCR and normalized by *β‐actin* for human cells or by *ribosomal protein S15* (*RpS15*) for mouse cells as internal standard. Data are expressed as mean ± SE of the samples (*n* = 4–6). Statistical analyses were performed using Student's *t*‐test. IH significantly increased the mRNA levels of *IL‐8*, *ON*, and *MN* in both muscle (RD and C2C12) cells. *GLUT4*, *MAPK14*, *PI3KR2*, *SIRT2*, *TNFα*, *IL‐6*, *IL‐15*, *IRISIN*, and *DCN* mRNAs levels in IH‐stimulated RD cells were 1.104, 0.9821, 1.007, 1.044, 0.8798, 1.020, 1.099, 1.031, and 1.031‐folds against normoxia (*p* = 0.1359, 0.6857, 0.5248, 0.5073, 0.3248, 0.8539, 0.1328, 0.2225, and 0.8294, respectively). The mRNA levels of *Tnfα*, *Il‐6*, *Mstn*, and *Irisin* in IH‐stimulated differentiated C2C12 cells were 1.185, 0.5283, 0.6559, and 0.7994‐folds (*p* = 0.5533, 0.3395, 0.6559, and 0.3952, respectively).

We exposed human RD muscle cells and differentiated mouse C2C12 muscle cells to normoxia or IH for 24 h. After the treatment, we measured the mRNA levels of *GLUT4*, *MAPK14*, *PI3KR2*, *SIRT2*, *TNFα*, *IL‐6*, *IL‐8*, *IL‐15*, *MSTN*, *BDNF*, *IRISIN*, *DCN*, *SPARC*/*ON*, and *ERFE*/*MN* by real‐time reverse transcriptase‐polymerase chain reaction (RT‐PCR). As shown in Figure 1A, IH significantly increased the mRNA levels of *MSTN*, *BDNF*, *IL‐8*, *ON*, and *MN* in the human RD muscle cells, whereas IH‐specific elevations were not observed in *GLUT4*, *MAPK14*, *PI3KR2*, *SIRT2*, *TNFα*, *IL‐6*, *IL‐15*, *IRISIN*, and *DCN*. In mouse C2C12 cells that have differentiated into muscle cells, IH significantly increased the mRNA levels of *IL‐8*, *On*, and *Mn*, but no IH‐specific elevations in *Mstn* and *Bdnf* were observed (Figure 1B).

**FIGURE 2:**
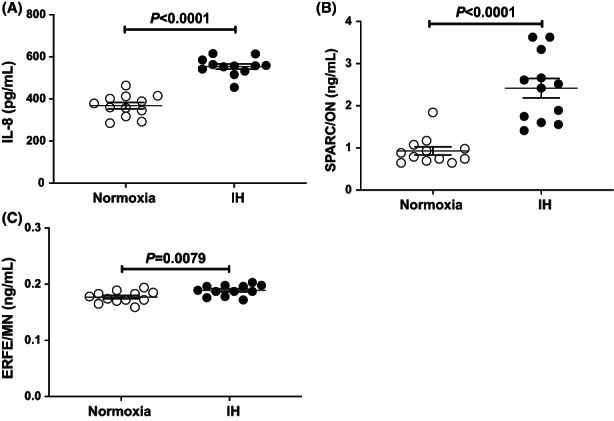
Concentrations of IL‐8, ON, and MN in RD muscle cell culture medium. RD cells were treated with normoxia or IH for 24 h. The (A) IL‐8, (B) ON, and (C) MN concentrations in the medium were measured by ELISA. Data are expressed as mean ± SE for each group (*n* = 12). Statistical analyses were performed using Student's *t*‐test.

Furthermore, we measured the IL‐8, ON (SPARC), and MN (ERFE) protein levels in the RD cell culture medium by using enzyme‐linked immunosorbent assay (ELISA), and found that the levels of IL‐8, ON, and MN were significantly increased by IH: IL‐8 (*p* < 0.0001), ON (*p* < 0.0001), and MN (*p* = 0.0079) (Figure 2).

### 3.2 Regions essential for the IH‐induced IL‐8, ON, and MN promoter activities are localized

**FIGURE 3:**
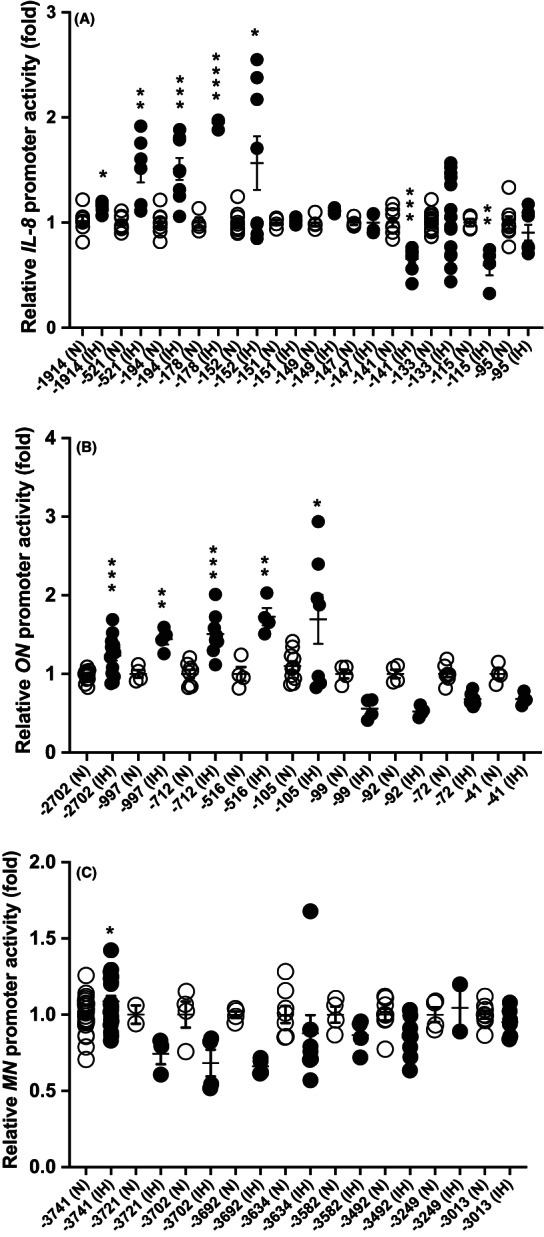
Localization of the essential region for IH‐induced *IL‐8*, *ON*, and *MN* promoter activities. RD cells were transfected with constructs containing various lengths of promoter fragments upstream of a firefly luciferase reporter gene in the pGL4.17 vector. After treatment with normoxia (N) or IH, the luciferase activity was measured. The diagram represents relative luciferase activities of (A) “‐1914” in *IL‐8*, (B) “‐2702” in *ON*, and (C) “‐3741” in *MN* in the normoxia group. All data are expressed as the mean ± SE for each group (*n* = 4–16). *: *p* < 0.05 vs Normoxia, **: *p* < 0.01 vs Normoxia, ***: *p* < 0.001 vs Normoxia, and ****: <*p* < 0.0001 vs Normoxia. Statistical analyses were performed using Student's *t*‐test.

In order to investigate the mechanism by which the mRNA levels of *IL‐8*, *ON*, and *MN* were upregulated by IH, we prepared the reporter plasmids by inserting various lengths of *IL‐8*, *ON*, and *MN* promoter fragments upstream of a firefly luciferase reporter gene in the pGL4.17 vector, transfected them into RD cells, and measured their luciferase activities after IH treatment. As shown in Figure 3A, a deletion down to position −152 of the *IL‐8* promoter region resulted in the IH‐induced upregulation of the reporter gene expression, but an additional deletion to nucleotide −151 attenuated the IH‐induced promoter activity. Concerning *ON* promoter, the deletion down to position −105 caused the IH‐induced upregulation of the reporter gene expression, but an additional deletion to nucleotide −99 attenuated the IH‐induced promoter activity (Figure 3B). Similarly, a deletion down to position −3741 of the *MN* promoter caused the IH‐induced upregulation of the reporter gene expression, but an additional deletion to nucleotide −3738 attenuated the IH‐induced promoter activity (Figure 3C). These results indicate that the IH‐induced upregulation of *IL‐8*, *ON*, and *MN* mRNAs is caused by the transcriptional activation of *IL‐8*, *ON*, and *MN* genes and that the −152 to −151 promoter region of the *IL‐8* gene, −105 to −99 region of the *ON* gene, and the −3741 to −3738 region of the *MN* gene are essential for the IH‐induced promoter activity.

### 3.3 OCT1 and NRF2 are key factors for the IH‐induced upregulation of IL‐8, ON, and MN mRNA expression levels

To further investigate the mechanism by which IH upregulates the *IL‐8*, *ON*, and *MN* mRNA levels, we conducted a computer‐aided search for sequences similar to known *cis*‐acting elements containing the −152 to −151 promoter region of the *IL‐8* gene, the −105 to −99 promoter region of the *ON* gene, and the −3741 to −3738 region of the *MN* gene using the TFBIND program (http://tfbind.hgc.jp). The result showed that the −152 ~ promoter region of *IL‐8* and the −3741 ~ promoter region of *MN* contained the possible OCT1 binding sequences, whereas the −151 ~ promoter region of *IL‐8* and the −3738 ~ promoter region of *MN* lost the OCT1 binding sequences. The −105 ~ promoter region of *ON* contains a possible NRF2 transcription factor binding sequence, whereas there is no NRF2 binding sequence in the −99 ~ promoter region.

**FIGURE 4:**
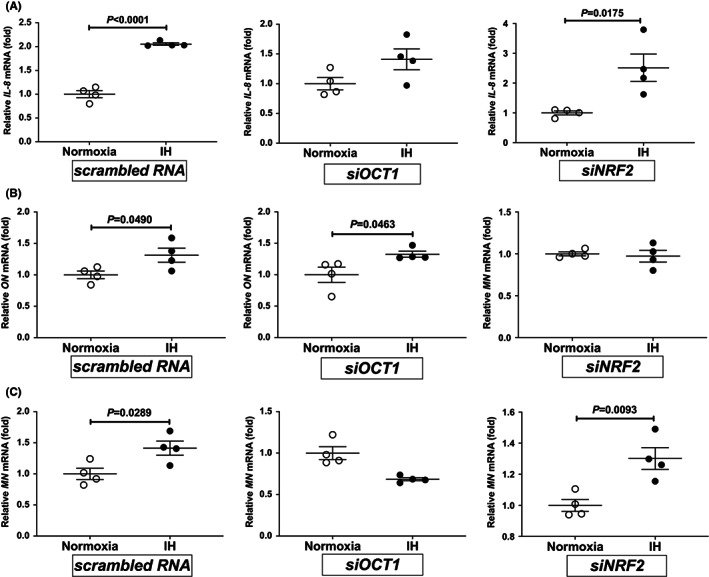
Inhibition of the IH‐induced upregulation of the *IL‐8*, *ON*, and *MN* mRNAs by transfection of OCT1 and NRF2 siRNAs into RD cells. After the introduction of OCT1 and NRF2 siRNAs, RD cells were treated with normoxia or IH for 24 h. The mRNA expression levels of (A) *IL‐8*, (B) *ON*, and (C) *MN* were measured by real‐time RT‐PCR and normalized by β‐Actin as internal standard. Data are expressed as the mean ± SE for each group (*n* = 4). Statistical analyses were performed using Student's *t*‐test. IH‐induced expressions of *IL‐8* mRNA in siOCT1 introduction, of *ON* mRNA in siNRF2 introduction, and of *MN* mRNA in siOCT1 introduction were not upregulated (1.409, 0.972, and 0.6851‐fold, respectively).

**FIGURE 5:**
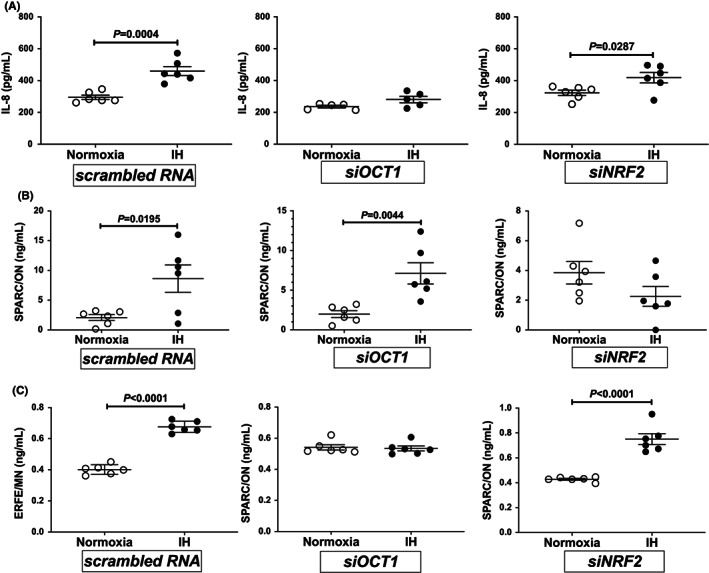
Concentrations of IL‐8, ON, and MN in RD muscle cell culture medium. After introduction of OCT1 and NRF2 siRNA, RD cells were treated with normoxia or IH for 24 h. The (A) IL‐8, (B) ON, and (C) MN concentrations in the medium were measured by ELISA. Data are expressed as mean ± SE for each group (*n* = 5–6). Statistical analyses were performed using Student's *t*‐test. IH‐induced expressions of IL‐8 in siOCT1 introduction, ON in NRF2 introduction, and MN in siOCT1 introduction were not upregulated (*p* = 0.0773, 0.1465, and 0.7654, respectively).

To investigate whether OCT1 and/or NRF2 were essential for the IH‐induced upregulation of *IL‐8*, *ON*, and *MN* mRNA levels, we introduced small interfering RNA (siRNA) s against human *OCT1* and *NRF2* mRNAs into RD cells and analysed the IH‐induced mRNA expression levels of *IL‐8*, *ON*, and *MN* by real‐time RT‐PCR. As shown in Figures 4A–C, the IH‐induced upregulation of *IL‐8*, *ON*, and *MN* mRNAs, respectively, were abolished by the human *OCT1* and *NRF2* siRNAs. Furthermore, IH‐induced upregulation of IL‐8, ON, and MN in the culture medium were also abolished by the introduction of human *OCT1* and *NRF2* siRNAs (Figures 5A–C). Specifically, the results indicated that OCT1 is a key factor for the IH‐induced upregulation of *IL‐8* and *MN* mRNA expression levels and that NRF2 serves as an essential factor for the IH‐induced upregulation of *ON* mRNA expression.

## 4 DISCUSSION

In this study, we demonstrated that IH exposure induced the increase in the *IL‐8*, *ON*, and *MN* mRNA levels in muscle cells. Furthermore, we elucidated the mechanisms by which IH upregulates the mRNA levels of the myokines *IL‐8*, *ON*, and *MN*, and we revealed the OCT1‐ and NRF2‐mediated transcriptional regulation in IH‐stimulated myocytes.

The causal mechanisms mediating the association between IH and insulin resistance/glucose intolerance are not well established; however, augmented dysfunction/inflammation in muscle cells might be involved.^18^, ^26^, ^27^, ^28^, ^29^, ^30^ It is well known that macrophages, which infiltrate into muscle tissue, are increased in obese patients, resulting in the upregulation of pro‐inflammatory cytokines, such as IL‐8.^31^ Some mechanisms linking IH stress and muscle tissue inflammation have been reported.^32^, ^33^ Recently, IH was shown to induce impairment of muscle tissue, leading to various changes in the secretion of inflammatory cytokines called myokines.^34^ Myokines, which are bioactive mediators produced and released by myocytes, play important roles in many physiological and pathophysiological processes that contribute in the modulation of homeostasis, lipid and/or glucose metabolism, and inflammation.^18^, ^35^, ^36^ IL‐8, also referred to as chemokine (C‐X‐C motif) ligand 8, is a key regulator of monocyte infiltration into skeletal muscle and is involved in muscle tissue inflammation and insulin resistance.^37^, ^38^, ^39^ In this study, the mRNA levels of *IL‐8* were significantly increased in the IH condition in mouse C2C12 and human RD muscle cells. ON, also referred to as SPARC, is an acidic extracellular matrix glycoprotein that plays a vital role in bone mineralization, cell‐matrix interactions, and collagen binding.^40^ ON plays a key role in obesity‐related insulin resistance, and increased levels of ON contribute to impaired glucose homeostasis; however, the role of ON as a myokine in the IH condition has not yet been fully elucidated.^41^, ^42^ In this study, ON was produced/secreted by C2C12 and RD myocytes in the IH condition. In SAS patients, the circulating levels of IL‐8 were reportedly elevated,^43^ and the production of IL‐8 in monocytes was upregulated.^44^ ON has also been reported to be associated with insulin resistance, dyslipidaemia, and inflammation.^42^ Overexpression of ON was observed in diet‐induced obese rats, and ON caused insulin resistance in 3 T3‐L1 mouse adipocytes.^45^

MN, also referred to as erythroferrone (ERFE) or C1q/TNF‐related protein isoform 15 (CTRP15), is a pro‐inflammatory myokine and reported to be associated with insulin resistance.^19^, ^46^ Recently, MN was proposed as a marker of insulin resistance, obesity, and diabetes^19^; however, the relationship between insulin resistance and MN expression remains controversial.^19^, ^47^ Our results demonstrated that MN was produced/secreted by RD myocytes in the IH condition (Figures 1 and 2). In addition, recent studies indicated that *MN* gene knockout female mice had larger gonadal fat pads and had developed mild insulin resistance when fed with a high‐fat diet, and *MN*‐deficient male mice showed impaired lipid tolerance.^48^ On the basis of our results and of the reported findings, *MN* may be upregulated in SAS patients and can lead these patients to develop insulin resistance/Type 2 diabetes and dyslipidaemia.^49^ In addition to *IL‐8* and *ON*, *MN* is possibly one of the myokines that promote insulin resistance in IH condition. There are some reports that upregulated myokines (IL‐8, ON, and MN) in muscle cells under insulin‐resistant conditions. Anti‐diabetic 5′ AMP‐activated protein kinase (AMPK) activator 5‐aminoimidazole‐4‐carboxamide‐1‐beta‐d‐ribonucleoside (AICAR) reduced IL‐6 and IL‐8 in human skeletal muscle cells.^50^ In insulin‐resistant condition, ON was upregulated in skeletal muscle cells via microRNA‐29a downregulation mechanism.^51^ Plasma levels of MN are associated with insulin resistance in adult humans.^52^ Therefore, IL‐8, ON, and MN expressed in skeletal muscle cells could be involved in IH‐induced diabetes and/or insulin resistance.

We investigated the mechanisms by which IH upregulates the mRNA levels of *IL‐8*, *ON*, and *MN*, and we found that the promoter activities of these genes were increased by IH via OCT1 and NRF2. This finding suggests that the IH‐induced upregulation of *IL‐8*, *ON*, and *MN* mRNAs is regulated in the transcriptional step.

Interestingly, significant increases in the *IL‐8*, *ON*, and *MN* gene expression levels induced by IH were observed. The subsequent promoter assays indicated that the IH‐induced upregulation of *IL‐8*, *ON*, and *MN* mRNAs was caused by the transcriptional activation of these genes. In addition, RNA interference experiments revealed that the transcriptional activation of *IL‐8* and *MN* by IH required OCT1 and that the transcriptional activation of *ON* by IH needed NRF2. Furthermore, we demonstrated that both OCT1 and NRF2 are essential for the IH‐induced upregulation of *IL‐8*, *MN*, and *ON* mRNA expression levels. The gene expression levels of *IL‐8*, *MN*, and *ON*, which are expressed in muscle cells, are remarkably upregulated by IH through the transcription factors OCT1 and NRF2. As a computer‐aided search revealed that the −152 to −151 region of the *IL‐8* promoter and the −3741 to −3738 region of the *MN* promoter, as well as the −105 to −99 region of the *ON* promoter, (both promoters are essential for the IH‐induced *IL‐8*, *MN*, and *ON* transcription) include the possible OCT1 and NRF2 transcription factor binding sequences; thus, we focused on OCT1 and NRF2 as important players in the IH‐induced upregulation of *IL‐8*, *MN*, and *ON* mRNAs in muscle cells.

OCT1, a POU class 2 homeobox1 (POU2F1), transcription factor, was among the first identified members of the POU transcription factor family.^53^ The members of this family contain the POU domain, a 160‐amino acid region necessary for DNA binding to the octameric sequence ATGCAAAT.^54^ Oct1 was reported to function as a sensor for metabolic and stress signals in pancreatic islets.^55^ Although OCT1 expression has not yet been reported in SAS patients or in cells in the IH condition, OCT1 could function as an intramuscular IH sensor to express IL‐8 and MN.

NRF2, which was originally isolated as a homologue of the haematopoietic transcription factor NF‐E2 p45,^56^ is the key transcription factor regulating antioxidant response. NRF2 signalling is repressed by Kelch‐like ECH‐associated protein 1 (KEAP1)^57^ at basal condition and is induced by oxidative stress. Most papers reporting on the relationship between NRF2 and SAS/IH suggested some protective functions of NRF2 in SAS/IH conditions.^58^, ^59^, ^60^, ^61^ Stress‐induced upregulation of ON was reported in vascular smooth muscle cells.^62^, ^63^ It is quite reasonable that the KEAP1‐NRF2 system functions as an intracellular sensor for oxygen and oxidative stress in IH condition. As a result, *ON* transcription could be activated by NRF2.

In this study, we observed no elevation of *GLUT4* mRNA in IH‐treated human RD cells (Figure 1A). Siquws et al. exposed rats to intermittent hypoxia (IH) for 30 days and reported that there is no change in the expressions of Glut1 and Glut4 in soleus muscle but an increase in the translocation of Glut4 from vesicles to the plasma membrane.^64^ In contrast, Wang et al. exposed rat to IH for 28 days and reported that the expressions of *Glut4* mRNA, total Glut4, and plasma membrane protein of Glut4 in skeletal muscle were decreased.^65^ Thus, the effect of IH on Glut4 expression and localization seems to be controversial. *Bdnf* mRNA was elevated in IH and further elevated in SH in human RD and mouse C2C12 cells (Figure 1A,B). Although there is no report concerning Bdnf elevation in IH condition, Bdnf is reported to be a positive‐acting myokine that is abundantly expressed in slow‐twitch skeletal muscle fibres, and its beneficial effects in skeletal muscle are mediated through AMPK‐PGC1α‐mediated mitochondrial function and β‐oxidation.^66^, ^67^, ^68^, ^69^ In SH condition, Nagahisa and Miyata reported that the muscle fibre area was decreased and mRNA expression of Bdnf was significantly increased in young soleus muscle.^70^ Therefore, in stress condition (SH and/or IH), it is quite possible that muscle cells express Bdnf to maintain muscle function and cell numbers. Although the mechanism of hypoxia‐induced Bdnf expression is interesting, it is not IH‐specific phenomenon (Figure 1B) and therefore we did not further investigated in this paper.

In this study, we use in vitro IH model to investigate the change in gene expression and its molecular mechanism. How similar/different to/from SAS patients are problems in in vitro system. Although the O_2_ concentrations of in vitro system is similar to IH patients and the in vitro system can examine direct effects of IH excluding effects of other organs and cells,^71^ the results from in vitro system sometimes are different from that occur in SAS patients. Therefore, our results obtained in this study indicated a possible occurrence in SAS patients and clinical studies using SAS patients are required.

In conclusion, this study revealed that the gene expression levels of *IL‐8*, *ON*, and *MN* in IH‐treated myocytes were increased by OCT1, which acts on the −152 to −151 region of the *IL‐8* promoter and on the −3741 to −3738 region of the *MN* promoter, and by NRF2, which acts on the −105 to −99 region of the *ON* promoter. Our results suggest that in SAS patients, the upregulation of *IL‐8*, *ON*, and *MN* may induce a pro‐inflammatory phenotype in muscle tissue, leading to the development of insulin resistance and decreased insulin sensitivity.

## AUTHOR CONTRIBUTIONS

**Shin Takasawa:** Conceptualization (lead); funding acquisition (equal); investigation (lead); methodology (lead); project administration (lead); writing – original draft (lead). **Ryogo Shobatake:** Conceptualization (supporting); data curation (supporting); funding acquisition (equal); investigation (supporting); validation (supporting); writing – review and editing (supporting). **Asako Itaya‐Hironaka:** Data curation (supporting); methodology (supporting); writing – review and editing (supporting). **Mai Makino:** Data curation (supporting); writing – review and editing (supporting). **Tomoko Uchiyama:** Data curation (supporting); writing – review and editing (supporting). **Sumiyo Sakuramoto‐Tsuchida:** Data curation (supporting); writing – review and editing (supporting). **Yoshinori Takeda:** Data curation (supporting); writing – review and editing (supporting). **Hiroyo Ota:** Data curation (supporting); funding acquisition (equal); writing – review and editing (supporting). **Akiyo Yamauchi:** Data curation (supporting); writing – review and editing (supporting).

## CONFLICT OF INTEREST

The authors confirm that there are no conflicts of interest.

## Data Availability

The data are available on request from the authors.

## References

[1] Kryger MH . Diagnosis and management of sleep apnea syndrome. Clin Cornerstone. 2000;2:39‐47. doi:10.1016/s1098-3597(00)90039-5 10875045

[2] Dempsey JA , Veasey SC , Morgan BJ , O'Donnell CP . Pathophysiology of sleep apnea. Physiol Rev. 2010;90:47‐112. doi:10.1152/physrev.00043.2008 20086074 PMC3970937

[3] Peppard PE , Young T , Barnet JH , Palta M , Hagen EW , Hla KM . Increased prevalence of sleep‐disordered breathing in adults. Am J Epidemiol. 2013;177:1006‐1014. doi:10.1093/aje/kws342 23589584 PMC3639722

[4] Ota H , Fujita Y , Yamauchi M , Muro S , Kimura H , Takasawa S . Relationship between intermittent hypoxia and type 2 diabetes in sleep apnea syndrome. Int J Mol Sci. 2019;20:4756. doi:10.3390/ijms20194756 31557884 PMC6801686

[5] Nannapaneni S , Ramar K , Surani S . Effect of obstructive sleep apnea on type 2 diabetes mellitus: a comprehensive literature review. World J Diabetes. 2013;4:238‐244. doi:10.4239/wjd.v4.i6.238 24379913 PMC3874482

[6] Rajan P , Greenberg H . Obstructive sleep apnea as a risk factor for type 2 diabetes mellitus. Nat Sci Sleep. 2015;7:113‐125. doi:10.2147/NSS.S90835 26491377 PMC4599645

[7] Nadeem R , Singh M , Nida M , et al. Effect of obstructive sleep apnea hypopnea syndrome on lipid profile: a meta‐regression analysis. J Clin Sleep Med. 2014;10:475‐489. doi:10.5664/jcsm.3690 24910548 PMC4046360

[8] Bradley TD , Floras JS . Obstructive sleep apnoea and its cardiovascular consequences. Lancet. 2009;373:82‐93. doi:10.1016/S0140-6736(08)61622-0 19101028

[9] Javaheri S , Javaheri S , Javaheri A . Sleep apnea, heart failure, and pulmonary hypertension. Curr Heart Fail Rep. 2013;10:315‐320. doi:10.1007/s11897-013-0167-3 24097114 PMC4111242

[10] Bucks RS , Olaithe M , Eastwood P . Neurocognitive function in obstructive sleep apnoea: a meta‐review. Respirology. 2013;18:61‐70. doi:10.1111/j.1440-1843.2012.02255.x 22913604

[11] Vaessen TJA , Overeem S , Sitskoorn MM . Cognitive complaints in obstructive sleep apnea. Sleep Med Rev. 2015;19:51‐58. doi:10.1016/j.smrv.2014.03.008 24846772

[12] Carotenuto M , Esposito M , Parisi L , et al. Depressive symptoms and childhood sleep apnea syndrome. Neuropsychiatr Dis Treat. 2012;8:369‐373. doi:10.2147/NDT.S35974 22977304 PMC3430390

[13] Wallace A , Bucks RS . Memory and obstructive sleep apnea: a meta‐analysis. Sleep. 2013;36:203‐220. doi:10.5665/sleep.2374 23372268 PMC3543053

[14] Raschke S , Eckel J . (2013) Adipo‐myokines: two sides of the same coin—mediators of inflammation and mediators of exercise. Mediators Inflamm. 2013;2013:320724. doi:10.1155/2013/320724 23861558 PMC3686148

[15] Carson BP . The potential role of contraction‐induced myokines in the regulation of metabolic function for the prevention and treatment of type 2 diabetes. Front Endocrinol (Lausanne). 2017;8:97. doi:10.3389/fendo.2017.00097 28512448 PMC5411437

[16] Graf C , Ferrari N . Metabolic health—the role of adipo‐myokines. Int J Mol Sci. 2019;20:6159. doi:10.3390/ijms20246159 31817641 PMC6941068

[17] Ciaraldi TP , Ryan AJ , Mudaliar SR , Henry RR . Altered myokine secretion is an intrinsic property of skeletal muscle in type 2 diabetes. PLoS One. 2016;11:e0158209. doi:10.1371/journal.pone.0158209 27453994 PMC4959771

[18] Garneau L , Aguer C . Role of myokines in the development of skeletal muscle insulin resistance and related metabolic defects in type 2 diabetes. Diabetes Metab. 2019;45:505‐516. doi:10.1016/j.diabet.2019.02.006 30844447

[19] Li K , Liao X , Wang K , et al. Myonectin predicts the development of type 2 diabetes. J Clin Endocrinol Metab. 2018;103:139‐147. doi:10.1210/jc.2017-01604 29161407

[20] Ota H , Tamaki S , Itaya‐Hironaka A , et al. Attenuation of glucose‐induced insulin secretion by intermittent hypoxia via down‐regulation of CD38. Life Sci. 2012;90:206‐211. doi:10.1016/j.lfs.2011.11.011 22154909

[21] Uchiyama T , Ota H , Itaya‐Hironaka A , et al. Up‐regulation of *selenoprotein P* and *HIP/PAP* mRNAs in hepatocytes by intermittent hypoxia via down‐regulation of miR‐203. Biochem Biophys Rep. 2017;11:130‐137. doi:10.1016/j.bbrep.2017.07.005 28955777 PMC5614699

[22] Uchiyama T , Itaya‐Hironaka A , Yamauchi A , et al. Intermittent hypoxia up‐regulates *CCL2*, *RETN*, and *TNFα* mRNAs in adipocytes via down‐regulation of miR‐452. Int J Mol Sci. 2019;20:1960. doi:10.3390/ijms20081960 31013606 PMC6515141

[23] Takeda Y , Itaya‐Hironaka A , Yamauchi A , et al. Intermittent hypoxia upregulates the *renin* and *Cd38* mRNAs in renin‐producing cells via the downregulation of miR‐203. Int J Mol Sci. 2021;22:10127. doi:10.3390/ijms221810127 34576290 PMC8466835

[24] Takasawa S , Shobatake R , Takeda Y , et al. Intermittent hypoxia increased the expression of DBH and PNMT in neuroblastoma cells via microRNA‐275‐mediated mechanism. Int J Mol Sci. 2022;23:5868. doi:10.3390/ijms23115868 35682548 PMC9180443

[25] Takasawa S , Tsuchida C , Sakuramoto‐Tsuchida S , et al. Upregulation of *REG IV* gene in human intestinal epithelial cells by lipopolysaccharide via downregulation of microRNA‐24. J Cell Mol Med. 2022;26:4710‐4720. doi:10.1111/jcmm.17498 35946046 PMC9443949

[26] Jun J , Polotsky VY . Metabolic consequences of sleep‐disordered breathing. ILAR J. 2009;50:289‐306. doi:10.1093/ilar.50.3.289 19506316 PMC5689472

[27] Mackenzie RWA , Watt P . A molecular and whole body insight of the mechanisms surrounding glucose disposal and insulin resistance with hypoxic treatment in skeletal muscle. J Diabetes Res. 2016;2016:6934937. doi:10.1155/2016/6934937 27274997 PMC4871980

[28] Czech MP . Insulin action and resistance in obesity and type 2 diabetes. Nat Med. 2017;23:804‐814. doi:10.1038/nm.4350 28697184 PMC6048953

[29] Archer AE , Von Schulze AT , Geiger PC . Exercise, heat shock proteins and insulin resistance. Philos Trans R Soc B. 2018;373:20160529. doi:10.1098/rstb.2016.0529 PMC5717529 29203714

[30] DiMenna FJ , Arad AD . Exercise as ‘precision medicine’ for insulin resistance and its progression to type 2 diabetes: a research review. BMC Sports Sci Med Rehabil. 2018;10:21. doi:10.1186/s13102-018-0110-8 30479775 PMC6251139

[31] Lee H , Song W . Exercise and mitochondria remodeling in skeletal muscle in type 2 diabetes. J Obes Metab Syndr. 2018;27:150‐157. doi:10.7570/jomes.2018.27.3.150 31089557 PMC6504199

[32] Pourteymour S , Eckardt K , Holen T , et al. Global mRNA sequencing of human skeletal muscle: search for novel exercise‐regulated myokines. Mol Metab. 2017;6:352‐365. doi:10.1016/j.molmet.2017.01.007 28377874 PMC5369209

[33] Lund M , Dahle MK , Timmerhaus G , et al. Hypoxia tolerance and responses to hypoxic stress during heart and skeletal muscle inflammation in atlantic salmon (*Salmo salar*). PLoS One. 2017;12:e0181109. doi:10.1371/journal.pone.0181109 28700748 PMC5507449

[34] Cheng W‐J , Liu X , Zhang L , et al. Chronic intermittent hypobaric hypoxia attenuates skeletal muscle ischemia‐reperfusion injury in mice. Life Sci. 2019;231:116533. doi:10.1016/j.lfs.2019.06.008 31173783

[35] Otaka N , Shibata R , Ohashi K , et al. Myonectin is an exercise‐induced myokine that protects the heart from ischemia‐reperfusion injury. Circ Res. 2018;123:1326‐1338. doi:10.1161/CIRCRESAHA.118.313777 30566056

[36] Ahima RS , Park H‐K . Connecting myokines and metabolism. Endocrinol Metab (Seoul). 2015;30:235‐245. doi:10.3803/EnM.2015.30.3.235 26248861 PMC4595346

[37] Das DK , Graham ZA , Cardozo CP . Myokines in skeletal muscle physiology and metabolism: recent advances and future perspectives. Acta Physiol (Oxf). 2020;228:e13367. doi:10.1111/apha.13367 31442362

[38] Vettor R , Milan G , Rossato M , Federspil G . Review article: adipokines and insulin resistance. Aliment Pharmacol Ther. 2005;22(Suppl. 2):3‐10. doi:10.1111/j.1365-2036.2005.02587.x 16225463

[39] Kobashi C , Asamizu S , Ishiki M , et al. Inhibitory effect of IL‐8 on insulin action in human adipocytes via MAP kinase pathway. J Inflamm (Lond). 2009;6:25. doi:10.1186/1476-9255-6-25 19709445 PMC2746203

[40] Zinicola M , Menta PR , Ribeiro BL , Boisclair Y , Bicalho RC . Effects of recombinant bovine interleukin‐8 (rbIL‐8) treatment on health, metabolism, and lactation performance in Holstein cattle III: administration of rbIL‐8 induces insulin resistance in bull calves. J Dairy Sci. 2019;102:10329‐10339. doi:10.3168/jds.2019-16336 31495622

[41] Rosset EM , Bradshaw AD . SPARC/osteonectin in mineralized tissue. Matrix Biol. 2016;52‐54:78‐87. doi:10.1016/j.matbio.2016.02.001 PMC5327628 26851678

[42] Kos K , Wilding JPH . SPARC: a key player in the pathologies associated with obesity and diabetes. Nat Rev Endocrinol. 2010;6:225‐235. doi:10.1038/nrendo.2010.18 20195270

[43] Xu L , Ping F , Yin J , et al. Elevated plasma SPARC levels are associated with insulin resistance, dyslipidemia, and inflammation in gestational diabetes mellitus. PLoS One. 2013;8:e81615. doi:10.1371/journal.pone.0081615 24349098 PMC3857203

[44] Ming H , Tian A , Liu B , et al. Inflammatory cytokines tumor necrosis factor‐α, interleukin‐8 and sleep monitoring in patients with obstructive sleep apnea syndrome. Exp Ther Med. 2019;17:1766‐1770. doi:10.3892/etm.2018.7110 30783447 PMC6364239

[45] Ke D , Kitamura Y , Lejtenyi D , Mazer B , Brouillette RT , Brown K . Enhanced interleukin‐8 production in mononuclear cells in severe pediatric obstructive sleep apnea. Allergy Asthma Clin Immunol. 2019;15:23. doi:10.1186/s13223-019-0338-1 31015845 PMC6469051

[46] Shen Y , Zhao Y , Yuan L , et al. SPARC is over‐expressed in adipose tissues of diet‐induced obese rats and causes insulin resistance in 3T3‐L1 adipocytes. Acta Histochem. 2014;116:158‐166. doi:10.1016/j.acthis.2013.06.004 23910024

[47] Toloza FJK , Mantilla‐Rivas JO , Pérez‐Matos MC , et al. Plasma levels of myonectin but not myostatin or fibroblast‐derived growth factor 21 are associated with insulin resistance in adult humans without diabetes mellitus. Front Endocrinol (Lausanne). 2018;9:5. doi:10.3389/fendo.2018.00005 29445355 PMC5797732

[48] Gagliano‐Jucá T . Letter to the editor: “Myonectin predicts the development of type 2 diabetes”. J Clin Endocrinol Metab. 2018;103:1649. doi:10.1210/jc.2017-02542 29365119

[49] Little HC , Rodriguez S , Lei X , et al. Myonectin deletion promotes adipose fat storage and reduces liver steatosis. FASEB J. 2018;33:8666‐8687. doi:10.1096/fj.201900520R PMC6593887 31002535

[50] Lihn AS , Pedersen SB , Lund S , Richelsen B . The anti‐diabetic AMPK activator AICAR reduces IL‐6 and IL‐8 in human adipose tissue and skeletal muscle cells. Mol Cell Endocrinol. 2008;292:36‐41. doi:10.1016/j.mce.2008.06.004 18606210

[51] Galimov A , Hartung A , Trepp R , et al. Growth hormone replacement therapy regulates microRNA‐29a and targets involved in insulin resistance. J Mol Med (Berl). 2015;93:1369‐1379. doi:10.1007/s00109-015-1322-y 26199111 PMC4661224

[52] Toloza FJK , Mantilla‐Rivas JO , Pérez‐Matos MC , et al. Plasma levels of nyonectin but not myostatin or fibroblast‐derived growth factor 21 are associated with insulin resistance in adult humans without diabetes mellitus. Front Endocrinol (Lausanne). 2018;9:5. doi:10.3389/fendo.2018.00005 29445355 PMC5797732

[53] Sturm RA , Cassady JL , Das G , Romo A , Evans GA . Chromosomal structure and expression of the human *OTF1* locus encoding the Oct‐1 protein. Genomics. 1993;16:333‐341. doi:10.1006/geno.1993.1194 8314572

[54] Zhao FQ . Octamer‐binding transcription factors: genomics and functions. Front Biosci (Landmark Ed). 2013;18:1051‐1071. doi:10.2741/4162 23747866 PMC4349413

[55] Wang P , Jin T . Oct‐1 functions as a sensor for metabolic and stress signals. Islets. 2010;2:46‐48. doi:10.4161/isl.2.1.10017 21099293

[56] Itoh K , Igarashi K , Hayashi N , Nishizawa M , Yamamoto M . Cloning and characterization of a novel erythroid cell‐derived CNC family transcription factor heterodimerizing with the small maf family proteins. Mol Cell Biol. 1995;15:4184‐4193. doi:10.1128/MCB.15.8.4184 7623813 PMC230657

[57] Itoh K , Wakabayashi N , Katoh Y , et al. Keap1 represses nuclear activation of antioxidant responsive elements by Nrf2 through binding to the amino‐terminal Neh2 domain. Genes Dev. 1999;13:76‐86. doi:10.1101/gad.13.1.76 9887101 PMC316370

[58] Wang Y , Chai Y , He X , et al. Intermittent hypoxia simulating obstructive sleep apnea causes pulmonary inflammation and activates the Nrf2/HO‐1 pathway. Exp Ther Med. 2017;14:3463‐3470. doi:10.3892/etm.2017.4971 29042934 PMC5639295

[59] Zhou S , Wang J , Yin X , et al. Nrf2 expression and function, but not MT expression, is indispensable for sulforaphane‐mediated protection against intermittent hypoxia‐induced cardiomyopathy in mice. Redox Biol. 2018;19:11‐21. doi:10.1016/j.redox.2018.07.014 30096613 PMC6086220

[60] Xu H , Wang J , Cai J , et al. Protective effect of lactobacillus rhamnosus GG and its supernatant against myocardial dysfunction in obese mice exposed to intermittent hypoxia is associated with the activation of Nrf2 pathway. Int J Biol Sci. 2019;15:2471‐2483. doi:10.7150/ijbs.36465 31595164 PMC6775312

[61] Lian N , Zhang S , Huang J , Lin T , Lin Q . Resveratrol attenuates intermittent hypoxia‐induced lung injury by activating the Nrf2/ARE pathway. Lung. 2020;198:323‐331. doi:10.1007/s00408-020-00321-w 31960166

[62] Farrokhi E , Samani KG , Chaleshtori MH . Oxidized low‐density lipoprotein and upregulated expression of osteonectin and bone sialoprotein in vascular smooth muscle cells. Lab Med. 2014;45:297‐301. doi:10.1309/LMUJWVQFW6CJMSOQ 25316660

[63] Farrokhi E , Samani KG , Chaleshtori MH , Tabatabaiefar MA . Effect of oxidized low density lipoprotein on the expression of *Runx2* and *SPARC* genes in vascular smooth muscle cells. Iran Biomed J. 2015;19:160‐164. doi:10.7508/ibj.2015.03.005 26025968 PMC4571011

[64] Siques P , Brito J , Flores K , et al. Long‐term chronic intermittent hypobaric hypoxia induces glucose transporter (GLUT4) translocation through AMP‐activated protein kinase (AMPK) in the soleus muscle in lean rats. Front Physiol. 2018;9:799. doi:10.3389/fphys.2018.00799 30002630 PMC6031730

[65] Wang X , Yu Q , Yue H , Zeng S , Cui F . Effect of intermittent hypoxia and rimonabant on glucose metabolism in rats: involvement of expression of GLUT4 in skeletal muscle. Med Sci Monit. 2015;21:3252‐3260. doi:10.12659/msm.896039 26503060 PMC4629628

[66] Yang X , Brobst D , Chan WS , et al. Muscle‐generated BDNF is a sexually dimorphic myokine that controls metabolic flexibility. Sci Signal. 2019;12:eaau1468. doi:10.1126/scisignal.aau1468 31409756 PMC7219567

[67] Balakrishnan R , Thurmond DC . Mechanisms by which skeletal muscle myokines ameliorate insulin resistance. Int J Mol Sci. 2022;23:4636. doi:10.3390/ijms23094636 35563026 PMC9102915

[68] Delezie J , Weihrauch M , Maier G , et al. BDNF is a mediator of glycolytic fiber‐type specification in mouse skeletal muscle. Proc Natl Acad Sci U S A. 2019;116:16111‐16120. doi:10.1073/pnas.1900544116 31320589 PMC6690026

[69] Yamanaka M , Tsuchida A , Nakagawa T , et al. Brain‐derived neurotrophic factor enhances glucose utilization in peripheral tissues of diabetic mice. Diabetes Obes Metab. 2007;9:59‐64. doi:10.1111/j.1463-1326.2006.00572.x 17199719

[70] Nagahisa H , Miyata H . Influence of hypoxic stimulation on angiogenesis and satellite cells in mouse skeletal muscle. PLoS One. 2018;13:e0207040. doi:10.1371/journal.pone.0207040 30408093 PMC6224099

[71] Ota H , Takasawa S , Yamauchi M , Yoshikawa M , Tomoda K , Kimura H . Intermittent hypoxia in pancreatic beta cells. Pancreat Disord Ther. 2015;5:S5. doi:10.4172/2165-7092.S5-004

